# A List of Colleges

**Published:** 1897-10

**Authors:** 


### A List of Colleges.

By reference to the pages at the front of this number, there will be seen a list of the dental colleges of the country. This list may not appear in every number of the journal, but sufficiently often, possibly every alternate number, to suit the convenience of every reader.

A large proportion of the members of the profession is not at all aware of the number of dental colleges in our country, nor where most of them are located, nor by whom conducted. This information should be in the mind, or within immediate reach of every progressive dentist in the country. We trust this roster may fully serve the purpose for which it is intended.

